# Nomenclature proposal to describe vocal fold motion impairment

**DOI:** 10.1007/s00405-015-3663-0

**Published:** 2015-06-03

**Authors:** Clark A. Rosen, Ted Mau, Marc Remacle, Markus Hess, Hans E. Eckel, VyVy N. Young, Anastasios Hantzakos, Katherine C. Yung, Frederik G. Dikkers

**Affiliations:** Department of Otolaryngology, University of Pittsburgh Voice Center, University of Pittsburgh, Pittsburgh, PA USA; Department of Otolaryngology-Head and Neck Surgery, University of Texas Southwestern Medical Center, Dallas, TX USA; Department of ORL-Head and Neck Surgery, Louvain University Hospital of Mont-Godinne, Yvoir, Belgium; Department of Voice, Speech and Hearing Disorders, University Medical Center Hamburg-Eppendorf, Hamburg, Germany; Abteilung Hals-, Nasen- u Ohrenkrankheiten, Klinikum Klagenfurt am Wörthersee, Klagenfurt, Austria; First Department of ORL-HNS of University of Athens, Hippocrateion General Hospital, Athens, Greece; Department of Otolaryngology, University of California at San Francisco, San Francisco, CA USA; Department of Otorhinolaryngology, Head and Neck Surgery, University Medical Center, University of Groningen, P.O. Box 30.001, 9700 RB Groningen, The Netherlands

**Keywords:** Nomenclature, Vocal fold motion, Vocal fold paralysis, Vocal fold paresis

## Abstract

The terms used to describe vocal fold motion impairment are confusing and not standardized. This results in a failure to communicate accurately and to major limitations of interpreting research studies involving vocal fold impairment. We propose standard nomenclature for reporting vocal fold impairment. Overarching terms of vocal fold immobility and hypomobility are rigorously defined. This includes assessment techniques and inclusion and exclusion criteria for determining vocal fold immobility and hypomobility. In addition, criteria for use of the following terms have been outlined in detail: vocal fold paralysis, vocal fold paresis, vocal fold immobility/hypomobility associated with mechanical impairment of the crico-arytenoid joint and vocal fold immobility/hypomobility related to laryngeal malignant disease. This represents the first rigorously defined vocal fold motion impairment nomenclature system. This provides detailed definitions to the terms vocal fold paralysis and vocal fold paresis.

## Introduction

Significant variability exists regarding the terminology used to describe vocal fold motion impairment (no or reduced motion) in the literature and amongst the medical community. Terms such as vocal fold palsy, vocal fold immobility, vocal fold paralysis, vocal fold paresis, and hemilaryngeal palsy are at times used interchangeably or at other times, the same term is used to represent different findings amongst clinicians. This confusion is present for both unilateral vocal fold motion abnormalities as well as bilateral vocal fold motion problems. As a result of the lack of consensus on specific nomenclature, communication with patients and between clinicians is impaired. In addition, research findings from different institutions are difficult to interpret or compare due to this confusion in terms. The establishment of precise nomenclature to describe vocal fold motion impairment will lead to a better understanding of the patient’s clinical picture and facilitate future research in the field of vocal fold motion impairment.

The  authors recognize the need for a defined nomenclature to describe vocal fold motion abnormality. It is beyond the purview of this article to discuss the diagnostic criteria for each term in detail. This paper will also not discuss the topics of inappropriate vocal fold motion that can occur in certain conditions, such as paradoxical vocal fold motion disorder or vocal cord dysfunction [1, 2]. In addition, this paper will not discuss hyperkinetic vocal fold motion diseases such as spasmodic dysphonia or essential tremor of the larynx. Furthermore, the focus of this project is limited to the true vocal fold(s) and not supraglottic structures. What follows are definition of terms which we advocate to be used for description of vocal fold motion impairment: vocal fold immobility, vocal fold paralysis, vocal fold hypomobility and vocal fold paresis. We provide definitions and descriptions for each of these terms.

## Definition and assessment of vocal fold motion

Descriptions of vocal fold motion typically reflect motion of the full length of the vocal fold, rather than the individual components (i.e., cartilaginous vs. membranous). It is often best to focus one’s attention on vocal fold motion at the location of the vocal process of the arytenoid cartilage during adductory or abductory tasks (phonation, cough, Valsalva, sniffing, inspiration…). For this proposal, vocal fold motion also strictly refers to only motion of the vocal fold (level of the glottis) and thus, does not include motion of any aspect of the supraglottis (false vocal fold, petiole nor supraglottic portion of the arytenoid cartilages). In some cases of vocal fold immobility, the arytenoid is tilted anteriorly making visualization of the vocal process difficult. In these situations the movement (or lack thereof) of the posterior membranous vocal fold should be used to assess the vocal fold mobility status. Often the laryngeal examination can be recorded and reviewed to look at various details. If slight or minimal motion is only seen on moving image playback (frame-by-frame review), then it does not meet the definition of substantive or gross vocal fold motion. This proposal is based on seeing the presence (or determining the absence) of purposeful movement of the vocal fold. Specifically, the gross motion of the vocal fold should be task appropriate (abduction with sniff and respiration and/or adduction with cough, phonation…). This definition does not involve the small movement of the vocal fold associated with respiration or the movement of the vocal fold from contralateral vocal fold contact. Movement of the vocal fold is completely different from movement of the “mucosal wave” of the vocal fold. Thus, none of the terms discussed in this proposal pertain to mucosal vibration or pliability that is typically assessed with stroboscopy (or high speed video). Misinterpretation of “vocal fold motion” can occur when the mucosa of the vocal fold is seen to “move” because of the Bernoulli effect during inhalation. This does not constitute substantive, purposeful vocal fold motion. Likewise, mucosal wave vibration seen during phonation (using stroboscopy) does not provide evidence of purposeful vocal fold motion.

Vocal fold motion determination can be made with a variety of laryngeal visualization methods (mirror, flexible or rigid endoscopes) on awake individuals, not involving vocal fold palpation (i.e., direct laryngoscopy). In addition, determination of the vocal fold motion status should be done with the patient relaxed and comfortable (flexible endoscopy may be better than rigid or mirror endoscopy for this) and involves gross motion  (visualization of purposeful vocal fold motion or an absence of such motion) seen during the actual exam. Flexible laryngoscopy has the vital benefit of allowing the patient to perform tasks of phonation and vegetative tasks (cough, laugh, respiration) in the most “natural” position. Per-oral approaches to laryngeal visualization using an angled telescope can give greater magnification and excellent optical quality but involves a relatively “un-natural” position (tongue protrusion) which may or may not create an examination artifact. Proponents of the latter examination technique appropriately argue that if vocal fold motion is normal on a per-oral examination then no further examination is required. However, if there is any abnormality of vocal fold motion (speed or range of motion) seen on a rigid exam, then these findings should be confirmed or discarded by a trans-nasal flexible laryngoscopy evaluation. Often patients will display different degrees and/or patterns of motion with different tasks. Judgment of motion or amount of motion should be observed with a variety of tasks, especially with the patient performing tasks such as alternating between /i/ and sniff or vegetative tasks (cough, laugh…). The best gross vocal fold motion that occurs consistently throughout the exam seen during any of the exam tasks should be used to make the final decision on motion (yes or no) and degree of motion impairment.

## Vocal fold motion impairment and etiology

A vocal fold is immobile if there is no active or voluntary adduction or abduction on clinical examination. A hypomobile vocal fold has reduced range and/or speed of motion on either adductory or abductory tasks. These two terms describe the qualitative physical exam finding of vocal fold motion and makes no assumption of the etiology, and thus the diagnosis, for the impaired motion. The terms, vocal fold immobility and vocal fold hypomobility, should be used when a definitive etiology for the motion impairment has not been established. The use of this terminology does not imply an idiopathic status of the vocal fold motion impairment because its use informs the reader that all possible causes of the vocal fold motion impairment have not yet been fully evaluated. These terms are the ideal terms for description of the results of the physical examination of the vocal folds (preferably via flexible laryngoscopy). When vocal fold hypomobility is seen on flexible laryngoscopy, further description of degree of vocal fold hypomobility (mild, moderate or severe), description of the speed of vocal fold motion (reduced, normal…) and/or the range of motion assessed (decreased or normal) can be used as subjective descriptors of the examiner’s physical exam findings. None of these descriptors  have been validated to date, but they do play an important role in the clinical description of the vocal fold motion assessment. The terms vocal fold paralysis and vocal fold paresis indicate a neurologic etiology of the vocal fold motion abnormality seen on physical examination.

### Neurogenic vocal fold motion impairment

A very common cause of vocal fold motion impairment cases are due to a neurogenic etilogy. The use of the term neurogenic implies an abnormality in either the central and/or peripheral nervous systems. This abnormality can occur anywhere from the brain to the neuro-muscular junction of the systems involved with vocal fold motion. A paralyzed vocal fold (vocal fold paralysis) is a vocal fold that is immobile due to a known or suspected neurogenic etiology (most commonly a recurrent laryngeal or vagus nerve injury). The paralyzed vocal fold shows absence of gross motion, although a small degree of arytenoid movement may be observed with contraction and release of the inter-arytenoid muscle during glottal tasks. The known or suspected neurogenic etiology can be established by clinical history, other signs of vagal dysfunction like velopharyngeal insufficiency; and/or by other related cranial nerve deficits or electrodiagnostic testing (laryngeal electromyography). Clinical history that supports a neurogenic etiology for vocal fold paralysis includes, but is not limited to:

- Onset of voice change coinciding with neck or chest surgery in the vicinity of the course of the ipsilateral recurrent laryngeal nerve (RLN) or vagus nerve, or non-surgical trauma to the same areas;
- Onset of voice change coinciding with surgery or non-surgical trauma to the skull base or brainstem;
- Lateral medullary cerebro-vascular accident (Wallenberg’s stroke).
- Onset of voice change coinciding with intubation/general anesthesia;
- Onset of voice change in the context of an upper respiratory infection (URI), where the voice remained poor after the other URI symptoms have resolved;
- Radiographic evidence of a mass impinging on the RLN or vagus nerve.

A patient who presents with an immobile vocal fold and has a related history of an appropriate antecedent event at the time of the voice change may be regarded to have a suspected vocal fold paralysis. The level of suspicion depends on the particular circumstance of the event (clinical history ± physical exam findings).

Electrodiagnostic testing via laryngeal electromyography (LEMG) can be used to confirm a neurogenic etiology for vocal fold paralysis and may provide important prognostic information and alter treatment plans [3, 4]. The precise role of LEMG in the assessment of vocal fold motion impairment and interpretation of LEMG findings are areas of active investigation and are beyond the scope of this document. When the vocal fold is immobile and the patient history is ambiguous, LEMG may be useful to distinguish between a mechanical  and a neurogenic  etiology. This situation is most common in the clinical scenario of sudden onset of dysphonia with an associated vocal fold immobility following orotracheal intubation (or use of a laryngeal mask airway) associated with non-head and neck or chest surgery [5, 6]. In this scenario, the vocal fold immobility could be due to a vocal fold paralysis from the ETT cuff or a mechanical injury to the crico-arytenoid joint associated with the intubation (see “Vocal fold motion impairment related to the mechanical impairment of the crico-arytenoid joint”) [7, 8]. In this clinical situation, an LEMG can assist with determining the etiology for the immobile vocal fold (neurogenic vs. mechanical).

Vocal fold paresis involves partial motion impairment due to a known or suspected neurogenic etiology. It demonstrates some, but not normal, preservation of gross vocal fold mobility. A paretic vocal fold can have motion abnormalities in that either the range of motion and/or the speed of vocal fold motion is reduced. The most obvious cases are seen when the vocal fold motion abnormality is judged abnormal relative to that of the contralateral vocal fold.

We believe that a definition of vocal fold paresis based on impaired motion (including aspects of range of motion and speed of movement) is relatively straightforward and therefore clinically useful. This is frequently seen when upon follow-up of vocal fold paralysis patients laryngeal examination reveals gross recovery of vocal fold motion but not completely normal in the areas of range of motion and/or speed of movement. Many other laryngeal exam findings have been proposed to be associated with vocal fold paresis, including those based on the static appearance of the vocal fold (e.g., bowed appearance or reduced bulk), or based on stroboscopy findings: e.g., phase asymmetry, adduction of the contralateral ventricular fold, decreased false vocal fold tone with related increased “exposure” of the laryngeal ventricle, petiole deviation, and global laryngeal movement (e.g., rotation), as well as evidence of reduced laryngeal sensation. Since these laryngeal findings have to date unproven specificity for vocal fold paresis, we propose that the definition of paresis be used based only on observed motion impairment (hypomobility) with the appropriate neurogenic etiology history. While LEMG may confirm a neurogenic cause for the hypomobility, it may not be possible to establish a LEMG diagnostic threshold that separates normal from abnormal, rendering an electromyographic definition for paresis less useful [9].

The term vocal fold palsy has been previously used variably to describe both vocal fold paralysis and paresis. Palsy is a term that can be used to encompass the entire spectrum of neurogenic vocal fold motion impairment however because of its lack of clarity; we do not endorse the use of palsy in the context of vocal fold motion impairment.

### Vocal fold motion impairment related to the mechanical impairment of the crico-arytenoid joint

Mechanical causes of vocal fold motion impairment are far less common than neurogenic causes of vocal fold immobility (vocal fold paralysis) and typically involve a dysfunction of the crico-arytenoid (CA) joint. These can include dislocation/subluxation vs. fixation of the CA joint. These alterations of the CA joint may be related to trauma (both internal and external), neoplastic infiltration, extrinsic compression from large tumors, or inflammatory processes (such as CA joint arthritis/synovitis) [10–17]. Another cause of mechanical vocal fold motion impairment is scarring of the inter-arytenoid region (i.e., the soft tissue around the CA joint and the posterior commissure of the larynx [18]. This condition may be related to internal or external trauma [19]. Comparable to arthrogenic motion impairment, the diagnosis of vocal fold motion impairment secondary to scarring of the inter-arytenoid region may be confirmed with use of laryngeal electromyography (to rule out an underlying neurogenic etiology) and/or with palpation of the inter-arytenoid area [20–23]. Palpation of the inter-arytenoid area is best performed under either local anesthesia or general anesthesia without an endotracheal tube in place [21, 22].

### Vocal fold motion impairment related to laryngeal malignant disease

In esophageal, tracheal and bronchogenic cancer, vocal fold motion impairment can be readily attributed to recurrent laryngeal nerve infiltration and is hence neurogenic (vocal fold paralysis). Controversy exists if vocal fold hypomobility in the face of malignant disease along the path of the vagus or recurrent laryngeal nerve is a vocal fold paresis or not. This issue is beyond the scope of this document and requires further investigation to include malignant invasion as an etiology of vocal fold paresis. Vocal fold impairment secondary to laryngeal cancer may result from infiltration of the crico-arytenoid joint, infiltration of intra-laryngeal nerve supply, or from tumor masses infiltrating laryngeal muscles [24].

A differentiation between these causative factors is usually neither feasible nor germane to clinical care. Therefore, it seems appropriate to classify these lesions as a different entity (vocal fold immobility due to laryngeal malignant disease). In laryngeal cancer staging, vocal fold mobility is a component used for the classification of the primary tumor for glottic, supraglottic and subglottic carcinomas [25, 26].

- T1—tumors have normal mobility of the vocal cords.
- T2—tumors present with impaired vocal cord mobility.
- T3—tumors present with vocal cord fixation.

In this context, the terms “impaired mobility” and “fixation” have been poorly defined to date and leave room for interpretation. It is reasonable to “update” the terminology of the staging system by replacing “impaired vocal cord mobility” with vocal fold hypomobility and “vocal cord fixation” with vocal fold immobility.

## Proposed classification

This paper proposes that patients with vocal fold motion impairment should be described and classified (unilateral or bilateral condition) using these rigorously defined terms: vocal fold immobility, vocal fold hypomobility, vocal fold paralysis or vocal fold paresis. The terms of vocal fold immobility and hypomobility are global terms that are both physical exam descriptors and useful when the specific etiology of the vocal fold motion abnormality (neurogenic or mechanical) has not yet been determined. The terms vocal fold paralysis and vocal fold paresis denote a vocal fold motion abnormality (immobile and hypomobile, respectively) with a known or strongly suspected neurogenic etiology. In addition, the terms vocal fold immobility/hypomobility should be used when there is vocal fold abnormality in the context of a laryngeal malignancy (not malignancy along the path of the vagus or recurrent laryngeal nerve), vocal fold impairment (immobility or hypomobility) related to malignant laryngeal disease.

*Vocal fold immobility* An immobile vocal fold due to either a neurogenic, or mechanical limitation of the crico-arytenoid joint or laryngeal malignant disease

*Vocal fold hypomobility* A vocal fold that has purposeful, gross motion but is not normal in terms of motion, range of motion and/or speed. The etiology between neurogenic or mechanical or laryngeal malignant disease has not yet been determined

*Vocal fold paralysis* An immobile vocal fold due to a neurogenic etiology. The cause can be either due to central nervous system pathology (i.e., lateral medullary infarct) or peripheral nervous system abnormality (vagus or recurrent laryngeal nerve)

* Vocal fold paresis* A hypomobile vocal fold due to a neurogenic etiology

*Vocal fold immobility/hypomobility related to the mechanical impairment of the crico-arytenoid joint* Vocal fold motion impairment (immobile or hypomobile) due to either anatomic abnormality of crico-arytenoid joint (dislocation, fixation…) or scar tissue of the soft tissues of the posterior commissure (posterior glottis stenosis)

*Vocal fold motion immobility/hypomobility related to laryngeal malignant disease* Vocal fold motion impairment (immobile or hypomobile) due malignant invasion of the intrinsic larynx (intra-laryngeal neural, muscle or joint).

## Conclusion

This proposal represents the first system to provide detailed description and definitions of terms for vocal fold motion impairment. These terms now have a specific definition and can be used to facilitate physician education and research regarding patients with vocal fold motion impairment. This proposal is designed to be clinically useful and be appropriate to most but not all clinical scenarios. The proposed language will facilitate improved clinical and scientific communications in both oral and written descriptions of vocal fold motion impairment. We hope this proposal based on expert opinion will stimulate further discussion and research on this topic.
